# Supplementary material for: Usability of Mixed Reality for Naloxone Training: Iterative Development and Field Testing of ReviveXR
Source: Healthcare (Basel). 2025 Jun 17;13(12):1449. doi: 10.3390/healthcare13121449 (PMC12192816; doi:10.3390/healthcare13121449)
Supplement: Supplementary file 1 [file healthcare-13-01449-s001.zip › healthcare-3640830-supplementary.pdf]

Supplementary Table S1. Summary of Revisions Made to ReviveXR Based on Round 1 Feedback

No.	Requested Change	Description of Revision Implemented
		Content Feedback
1	Integration of Introductory Training Video	The content from the "ReviveXR Intro Video" was embedded directly into the training module. An AI-generated virtual instructor was incorporated to deliver the introductory guidance. The video now includes captions and emphasizes safety messaging, such as advising participants to step back after administering naloxone due to potential post-revival agitation.
2	Reduction of Rescue Breaths Count	The rescue breathing component was streamlined by reducing the number of breaths to three per cycle.
3	Removal of Initial Object Search	The object search feature at the beginning of the training was removed for clarity and flow. Only the naloxone device (Actual one) was retained, placed in a standardized location. Progression through the training no longer depends on searching for or collecting objects.
4	Enhancement of 911 Call Scenario	The 911 call segment was revised to simulate the experience of the participant using their own phone. Instructional dialogue was updated to state, "Now pick up your phone and dial 911," to reinforce user realism and engagement and no need actual dialing of 911 where it detects the movement to complete the step.
		Software feedback
1	Minimization of Glitches During Object Interaction	Object-related glitches—such as items falling unexpectedly or appearing incorrectly on the virtual patient—were addressed through improved object recognition and collision handling mechanisms.
2	Enhancement of Phone Appearance	The virtual phone interface was removed and instead update the instruction panel.
3	Automatic Patient Placement	The virtual patient was repositioned automatically with the participants movements at the start of the scenario. This adjustment removed the difficulty for participants to place the patient.
4	Instruction Box Glitches	Multiple bugs affecting the instruction panel were resolved, including yellow highlight overlays, lagging text, disappearing content, and video playback issues that required app restarts.
5	Feedback Mechanism for Participant Actions	Feedback mechanisms—both auditory and visual—were implemented to acknowledge user actions. When multiple steps were required in a single instruction panel, prompts were added to help users identify missed actions before progressing.
6	Improved Detection of Rescue Breathing Technique	The system was refined to better detect correct hand positioning and breath delivery. Inaccurate actions, such as simply closing mouths without breath delivery, no longer allowed progress to the next step.
7	Adaptive Instruction Panel Behavior	The instruction panel was programmed to follow the participant's field of view. It now disappears during action execution and reappears when the participant looks away, enhancing usability and reducing visual clutter.
8	Correction of Consciousness Testing Recognition	The program was updated to properly register the knuckle-to-chest stimulus as a valid method for checking consciousness. This interaction now triggers an appropriate response and is required before advancing.
